# Turbulent dynamics and whole-brain modeling: toward new clinical applications for traumatic brain injury

**DOI:** 10.3389/fninf.2024.1382372

**Published:** 2024-03-25

**Authors:** Noelia Martínez-Molina, Yonatan Sanz-Perl, Anira Escrichs, Morten L. Kringelbach, Gustavo Deco

**Affiliations:** ^1^Computational Neuroscience Group, Center for Brain and Cognition, Department of Information and Communication Technologies, Universitat Pompeu Fabra, Barcelona, Spain; ^2^Centre for Eudaimonia and Human Flourishing, Linacre College, University of Oxford, Oxford, United Kingdom; ^3^Department of Psychiatry, University of Oxford, Oxford, United Kingdom; ^4^Department of Clinical Medicine, Center for Music in the Brain, Aarhus University and The Royal Academy of Music Aarhus/Aalborg, Aarhus, Denmark; ^5^Institució Catalana de la Recerca i Estudis Avançats, Barcelona, Spain

**Keywords:** traumatic brain injury, nonlinear brain dynamics, turbulence, whole-brain modeling, neuroimaging biomarkers, stratified neurology

## Abstract

Traumatic Brain Injury (TBI) is a prevalent disorder mostly characterized by persistent impairments in cognitive function that poses a substantial burden on caregivers and the healthcare system worldwide. Crucially, severity classification is primarily based on clinical evaluations, which are non-specific and poorly predictive of long-term disability. In this Mini Review, we first provide a description of our model-free and model-based approaches within the turbulent dynamics framework as well as our vision on how they can potentially contribute to provide new neuroimaging biomarkers for TBI. In addition, we report the main findings of our recent study examining longitudinal changes in moderate-severe TBI (msTBI) patients during a one year spontaneous recovery by applying the turbulent dynamics framework (model-free approach) and the Hopf whole-brain computational model (model-based approach) combined with *in silico* perturbations. Given the neuroinflammatory response and heightened risk for neurodegeneration after TBI, we also offer future directions to explore the association with genomic information. Moreover, we discuss how whole-brain computational modeling may advance our understanding of the impact of structural disconnection on whole-brain dynamics after msTBI in light of our recent findings. Lastly, we suggest future avenues whereby whole-brain computational modeling may assist the identification of optimal brain targets for deep brain stimulation to promote TBI recovery.

## 1 Introduction: the turbulent dynamics framework and whole-brain computational modeling

As such, the physical phenomenon of turbulence in fluids has been an object of intense study in the scientific community for more than four centuries, starting from the insightful observations and meticulous drawings by Leonardo Da Vinci (1507) (Deco et al., 2021a) to the mathematical equations developed by Navier (1823) and eventually refined by Stokes (1843) in order to describe the turbulent regime at the microscopic level. However, as already noticed by Da Vinci, the important properties of turbulence are also to be found at the macroscopic level. One of these properties implies the effective and fast transfer of energy across fluids that Andrey Kolmogorov described in his pioneering phenomenological theory of turbulence (Kolmogorov, 1941a,b). In this ground-breaking work, Kolmogorov introduced the concept of structure functions, which allowed him to quantify the energy cascades that balance kinetics and viscous dissipation based on the correlations between two spatial points in a fluid and, as a result, demonstrate a power scaling law. Importantly, these power laws provide a mathematical foundation for Richardson's earlier concept of cascaded eddies (Richardson, 1922), i.e., a cascade of kinetic energy that is transferred from larger to smaller scales without loss in the so-called inertial subrange. At a more abstract level, effective energy transfer can be considered equivalent to efficient information processing, which makes the study of turbulence in the human brain particularly appealing as it requires the rapid integration of information across spatially distant regions.

To study turbulence in a non-hydrodynamic context such as the human brain, one needs to find an appropriate mathematical formalism. In this regard, Yoshiki Kuramoto's theory of coupled oscillators in the 1980s was crucial (Kuramoto, 1984). In fact, using coupled nonlinear oscillators, Kuramoto was able to describe turbulence in many different physical systems. Specifically, in the coupled oscillator framework, the Kuramoto local order parameter represents a spatial average of the complex phase factor of the local oscillators weighted by the coupling. Amplitude turbulence is then defined as the standard deviation of the modulus of the Kuramoto local order parameter and can be applied to the empirical data of any physical system, including the human brain. Interestingly, brain activity can be computationally modeled using Stuart-Landau coupled oscillators with a high level of accuracy, therefore providing some degree of convergence with turbulence as originated by Kuramoto's coupled oscillators. Taken together, this motivated the investigation of turbulence in the human brain by combining Kolmogorov's structure functions with Kuramoto's local order parameter as well as building a Hopf whole-brain model with Stuart-Landau oscillators to understand the causal mechanisms given rise to a turbulent regime.

The discovery that human brain activity is supported by turbulent dynamics was indeed recently made by using a high-quality large-scale resting-state dataset of 1,003 Human Connectome Project's participants (Deco and Kringelbach, 2020). In that study, we found that the whole-brain dynamics operates in a turbulent regime that follows a consistent power law for functional brain correlations in a broad spatial range similar to that shown by Kolmogorov in fluid dynamics and thus indicative of a cascade of information processing. More recently, using the same dataset, these findings have been extended by incorporating higher-order structure functions showing that out-of-equilibrium turbulent dynamics are based on the deviations from scale invariance within the phenomenological Kolmogorov's theory (Perl et al., 2023).

**Figure 1:**
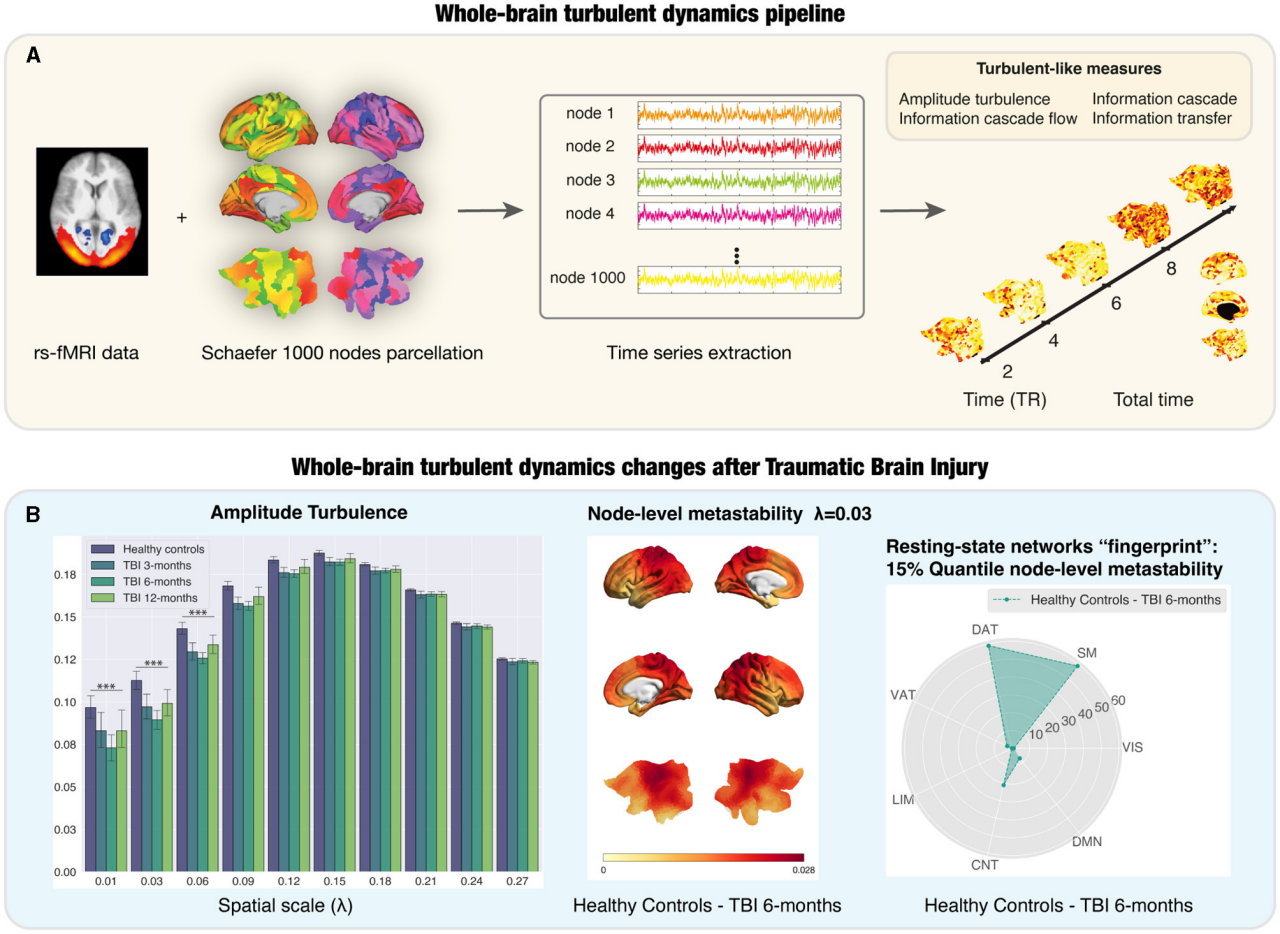
Whole-brain turbulent dynamics pipeline and its application to a longitudinal dataset of Traumatic Brain Injury (TBI) patients. **(A)** The implementation of the whole-brain turbulent dynamics framework requires resting-state functional MRI (rs-fMRI), typically using echo-planar images sampling the BOLD time course in each voxel in the brain. This is then combined with a parcellation scheme to recreate the regional time courses for each of the regions in the parcellation, in this case, the fine- grained Schaefer parcellation with 1,000 nodes (Schaefer et al., 2018). This allows for the extraction of regional time courses that are used to compute the analytic signal. The Kuramoto local order parameter can define the turbulent signatures of brain activity at different spatial scales in the vortex space. Four turbulent measures based on the Kuramoto local order parameter are calculated as potential neuroimaging biomarkers: amplitude turbulence, information cascade flow, information cascade and information transfer. **(B)** Left subpanel: Amplitude turbulence differs between TBI patients and healthy controls (HCs) at long distances in the brain over time, peaking at 6 months post-injury. Middle subpanel: Render brains representing the absolute difference of the node-level metastability between HCs and TBI patients at 6 months post-injury for scale λ= 0.03. Right subpanel: stage-specific resting-state networks “fingerprint” using radar plots for the number of nodes on the top 15% quantile of the absolute difference between HCs and TBI patients at 6-months post-injury comparison for λ= 0.03. Most of the nodes were ascribed to the SM, DAT, and CNT networks. TR: repetition time; LIM, limbic; CNT, control; DMN, default mode; DAT, dorsal-attention; VAT, ventral attention; VIS, visual; SM, somatosensory. Figure adapted with permission from Martínez-Molina et al. (2023).

An important additional consideration in the turbulent dynamics framework concerns the concept of the brain vortex space. In fluid dynamics, the vortices are essentially capturing the rotational kinetic energy. In contrast, the brain vortex space refers to the local level of synchronization at a given spatial scale across spacetime thus capturing the level of rotationality. Based on this central concept, we can define three additional measures to study different aspects of information propagation, namely information transfer, information cascade and information cascade flow. The information transfer indicates how the information travels across space at a specific spatial scale. The other two metrics are interrelated. The information cascade flow measures how the information travels from a given spatial scale to a lower scale in consecutive time steps. The information cascade is the average of the information propagation across spatial scales. It then follows that, by calculating these additional measures, we can provide a richer description of turbulent dynamics and information processing. Since this initial implementation, the turbulent dynamics framework (Figure 1A) has also helped to discriminate between different brain states in the healthy population and altered states of consciousness (De Filippi et al., 2021; Cruzat et al., 2022; Escrichs et al., 2022), suggesting that is a valid, sensitive and reliable measure. Noteworthily, this framework has also been adapted to direct measures of fast neural dynamics such as resting-state MEG (Deco et al., 2023).

The Hopf whole-brain modeling approach also allows to calculate other potential neuroimaging biomarkers based on the brain's reactivity to external *in silico* perturbations. Specifically, one can compute two additional measures: (i) susceptibility, the sensitivity of the whole-brain model to the processing of external stimulations, and (b) information capability, the standard deviation of the difference between the perturbed and unperturbed mean of the modulus of the Kuramoto local order parameter. In the context of turbulence, Deco and Kringelbach (2020) demonstrated that the optimal working point of the Hopf whole-brain model, i.e., showing the best fit to the empirical data, corresponded to a region of maximally developed amplitude turbulence. Remarkably, this was also the point where the information capability reached its maximum, further supporting the notion that amplitude turbulence is key for information processing. It is also important to note that, by incorporating the exponential distance rule (Ercsey-Ravasz et al., 2013) of anatomical connections as a cost-of-wiring principle, the Hopf whole-brain model showed an economy of anatomy that was able to reproduce turbulence. More recent refinements of this model have proved the benefits of adding the rare long-range connections found in the mammalian brain, which improved information processing (Deco et al., 2021b).

In this Mini Review, we first describe the findings from our recent study that point to potential neuroimaging biomarkers for TBI using a model-free and model-based approach within the turbulent dynamics framework. Next, we discuss how the model-based approach combined with a simulated attack in that study helped us to understand the effect of structural disconnection. Finally, we propose that deep brain stimulation treatments for TBI could benefit from presurgical assessment of clinical outcomes using whole-brain computational modeling.

## 2 Potential neuroimaging biomarkers in traumatic brain injury

Traumatic Brain Injury (TBI) poses a global burden on death and disability only second to cancer according to a recent estimate (National Academies of Sciences, Engineering, and Medicine, 2022), thus representing a significant public health concern. More specifically, recent reports provide an estimate of 50–60 million people with TBI per year worldwide (Feigin et al., 2013). TBI can result from diverse causes including falls, sports injuries, vehicle collisions, intimate partner violence, and military incidents affecting different age groups, from babies to the elderly (National Academies of Sciences, Engineering, and Medicine, 2022). Patients with TBI show a wide spectrum of symptoms, ranging from physical, behavioral and emotional to cognitive (Hoofien et al., 2001; Dikmen et al., 2009; Cantor et al., 2013; Wilde et al., 2022). This wide variation in the clinical manifestations of TBI is likely due to the complexity of the brain's organization, as well as to the patterns and extent of damage caused by external forces leading to TBI. In addition to focal brain damage, rapid acceleration and deceleration forces at the time of brain injury damage the axonal membrane resulting in the so-called diffuse axonal injury (DAI) (Martínez-Molina et al., 2022) that is thought to underlie alterations in brain network connectivity (Sharp et al., 2014). Moreover, DAI and neuroinflammation after TBI might influence the development of neurodegenerative disorders such as Alzheimer's disease (AD), which is a frequent late complication in these patients (Sharp et al., 2014). Given that impairment in executive functioning is one of the most common symptons after TBI, it is conceivable that DAI affects the long-range connections that shape the brain's information transmission capabilities across time and space (Deco et al., 2021b) and contribute to sustain critical dynamics in the presence of the slow information transfer between neurons (Deco et al., 2023).

Historically, patients with TBI have been classified into mild, moderate, and severe diagnostic categories based exclusively on clinical features such as the level of consciousness as evaluated with the Glasgow Coma Scale (GCS) (Teasdale and Jennett, 1974) or duration of post-traumatic amnesia (PTA) (Marshman et al., 2013). However, both the GCS score and the duration of PTA have been shown to be poorly reflective of pathophysiological mechanisms (King et al., 1997; Zuercher et al., 2009). This makes it clear that patients' stratification should be informed by objective measures of pathophysiology such as neuroimaging, neuroelectrophysiological and fluid biomarkers (Orešič et al., 2016; Majdan et al., 2017; Thelin et al., 2019). The discovery of new biomarkers could also help to better monitor the longitudinal progression of TBI and identify patients at high risk for developing neurodegenerative disease secondary to TBI. Although blood biomarkers are accessible cost-effective promising tools for TBI diagnosis and prognosis in primary care settings, they do not allow to assess the brain's abnormality patterns associated with TBI. A multi-modal approach that integrates neuroimaging biomarkers can circumvent this limitation and contribute to provide a more comprehensive understanding of the disease and its progression. Such an approach including biomarker panels combined with machine learning algorithms may help to identify specific injury profiles (Wilde et al., 2022). In this direction, some studies have started to explore the discriminatory power of a combination of fluid biomarkers in patients with suspected mild TBI with and without neuroimaging findings (Gill et al., 2018; Edwards et al., 2020).

Resting-state fMRI (rs-fMRI) data are increasingly being used in the development of new neuroimaging biomarkers for neurological and neuropsychiatric populations (Deco and Kringelbach, 2014) as these sequences have better signal-to-noise ratio compared to task-based studies, can be acquired in patients who may not be able to perform tasks and can be automatically preprocessed with currently available software tools (Whitfield-Gabrieli and Nieto-Castanon, 2012), which can facilitate their translation from research into clinical practice. According to the Food and Drug Administration (FDA), a biomarker can be defined as “a characteristic that is measured as an indicator of normal biological processes, pathogenic processes, or responses to an exposure or intervention, including therapeutic interventions” with the ideal requirements of a biomarker including being sensitive, specific, reproducible and operational among others (Wilde et al., 2022). In this regard, the turbulent dynamics framework holds great promise to provide new neuroimaging biomarkers as: (i) it can be easily computed from rs-fMRI data, (ii) captures global functional brain damage due to structural disconnection at multiple spatial scales, and (iii) reflects regional abnormalities when calculated at node-level providing specific “fingerprints” potentially useful for distinguishing between different subgroups of patients. This potential is very much in line with the possibility to combine the turbulent framework with the biotype approach, the latter being a data-driven strategy to identify clusters of patients (Brucar et al., 2023). In the field of computational neuropsychiatry, this approach has been used to cluster patients with major depressive disorder based on rs-fMRI data (Drysdale et al., 2017). Interestingly, the authors found different biotypes associated with specific clinical symptoms that showed a differential response to treatment with repetitive transcranial magnetic stimulation (rTMS), suggesting the predictive value of the biotype-based classification.

In addition to improved diagnosis, the turbulent dynamics framework could also provide prognostic biomarkers to characterize the longitudinal recovery trajectory after TBI. In our recent study (Martínez-Molina et al., 2023), we investigated the potential of the turbulent dynamics framework and whole-brain modeling to provide us with neuroimaging biomarkers that uncover TBI progression during one year of spontaneous recovery using a publicly available rs-fMRI dataset (Roy et al., 2017) with moderate-severe patients at the chronic stage. In our study (Figure 1B), we provided evidence of significantly reduced global amplitude turbulence in TBI patients at long distances in the brain, which, as mentioned above, suggests disruptions in the long-range connections that enhance information processing across time and space (Deco et al., 2021b). Node-level turbulence revealed specific resting-state networks “fingerprints” showing the difference between TBI patients and healthy controls (HCs), which could help to assess the neurobiological effectiveness of targeted treatments. Furthermore, we explored the behavioral relevance of these findings by analyzing the correlation between turbulent brain dynamics and a neuropsychological battery to evaluate executive function focusing on attention and working memory. Our results extended previous findings on the association between metastability and cognitive performance (Hellyer et al., 2015) by showing that, at baseline, working memory scores in TBI patients correlated with information cascade flow and amplitude turbulence in the default mode network at long distances. The results from the model-based approach showed a decrease in the global coupling strength in all time points when fitting the model of TBI patients (Martínez-Molina et al., 2023). TBI patients were also characterized by a *U*-shaped reduction in the susceptibility and information capability during the 1-year recovery trajectory.

This promising preliminary evidence could be further refined by examining the relationship between turbulent brain dynamics and genomic information, which would shed light on the neurobiological basis associated with disruptions in the turbulent regime with a particular focus on neuroinflammation. Indeed, the neuroinflammatory response to injury triggered by microglia activation after TBI can persist for months to years (Ramlackhansingh et al., 2011; Shitaka et al., 2011; Johnson et al., 2013). This persistent neuroinflammation might induce the propagation of abnormal proteins, and could be a causal factor in the subsequent neurodegeneration and further cognitive decline often seen after TBI (Gentleman, 2013). Recent studies have started to incorporate the gene expression profiles obtained from blood samples in Alzheimer's disease (AD) patients and investigated their relationship with neuroimaging biomarkers (Zhao et al., 2023). A gene-enrichment analysis revealed that the genes associated with a significant neuroimaging biomarker for AD were involved in immunity-related processes. Given the abovementioned neuroinflammatory response and risk for neurodegeration after TBI, future gene-enrichment analysis could help to ascertain whether genes associated with neuroinflammation and neurodegeneration might play a role in the underlying neurobiology captured by neuroimaging biomarkers obtained using turbulent dynamics and whole-brain modeling perturbation protocols.

## 3 Whole-brain models to evaluate the impact of focal lesions and for neurosurgical planning

By simulating normal spontaneous brain function, whole-brain computational modeling can provide a unique tool for understanding and predicting the impact of structural connectivity damage on brain dynamics and, more specifically, the effect of anatomical location and extent of the lesion (Alstott et al., 2009). Such an endeavor is particularly relevant to understand the functional consequences of lesions in stroke (Idesis et al., 2023) and TBI patients (Martínez-Molina et al., 2023), often presenting a heterogeneous pattern of lesions. Using the abovementioned longitudinal rs-fMRI dataset, we applied a simulated attack approach (Medaglia et al., 2022) to explore the influence of focal lesions on the brain's responsiveness to *in silico* perturbations of the whole-brain model (Martínez-Molina et al., 2023). In brief, the overlap between the patient's specific lesion mask and the parcellation scheme was calculated and used to create a group lesion mask to which we applied two structural disconnection methods: one weighted and one binary, the latter being more aggressive with a full deletion of the lesioned node's connectivity. Both approaches of structural disconnection applied to TBI patients led to decreased reactivity to external perturbations. Of note, the lowest values were found for the most aggressive binary approach. This indicates that, at the group level, the effects of lesions on the brain's reactivity are more prominent when there is a great degree of lesion overlap in the patients under study.

Although at present there is no gold standard treatment to promote the recovery of TBI-related cognitive impairments, deep brain stimulation (DBS) within the central lateral (CL) nucleus of the thalamus and the associated medial dorsal tegmental tract has been recently shown to improve executive function in six moderate-severe TBI patients at the chronic stage (Schiff et al., 2023). The CL thalamic neurons project to frontal and striatal regions and might contribute to reverse the cognitive deficits associated with disrupted frontostriatal connectivity after TBI (De Simoni et al., 2018). Despite the beneficial effects of the DBS therapy, there was considerable interindividual variability in the level of efficacy. In this regard, whole-brain computational modeling could help to predict the clinical outcomes of DBS presurgically in order to inform the decision to undergo such an invasive procedure. On the other hand, the selection of the stimulation target was based primarily on their previous work with a single patient in the minimally conscious state (Schiff et al., 2007) and non-human primates studies (Baker et al., 2016; Janson et al., 2021). While this is a scientifically valid approach, whole-brain computational modeling combined with individual structural connectivity could enable the exploration of the optimal brain targets for an individual patient. Although with less spatial resolution than implantable DBS, transcranial temporal interference stimulation (tTIS) (Grossman et al., 2017; Violante et al., 2023) stands as a non-invasive alternative that could also be used to electrically estimulate the striatum (Wessel et al., 2023) and might improve executive dysfunction in TBI patients with altered caudate connectivity (De Simoni et al., 2018). Regardless of the neurostimulation technique used, much more future research is needed before the translation of whole-brain modeling to the clinical setting for optimal brain targeting. In this sense, we envisage three main avenues for future work: (i) improve patient-specific whole-brain computational models to mitigate the impact of overfitting and measurement noise, (ii) study and predict the DBS- or tTIS-induced structural and functional changes as in van Hartevelt et al. (2014), and (iii) evaluate how well the whole-brain model is able to replicate the changes induced by DBS or tTIS after *in silico* stimulation of the same brain targets.

## 4 Conclusions

In summary, in this Mini Review, we have tried to show some progress in the discovery of neuroimaging biomarkers for TBI based on model-free and model-based approaches within the turbulent dynamics framework that have strong potential for application in future clinical practice and treatment trials. The complexity and heterogeneity of TBI calls for a combination of clinical variables and objective pathophysiological biomarkers to improve diagnosis—which to date relies primarily on non-specific clinical evaluations that poorly predict long-term disability—, prognosis and prediction of treatment efficacy. Furthermore, we have discussed how whole-brain computational models can increase our understanding of the effect of focal lesions and to identify optimal stimulation targets which, ultimately, can help to alleviate the long-term suffering associated with TBI.

## Author contributions

NM-M: Conceptualization, Project administration, Writing—original draft. YS-P: Methodology, Writing—review & editing. AE: Methodology, Writing—review & editing. MK: Conceptualization, Methodology, Writing—review & editing. GD: Conceptualization, Methodology, Supervision, Writing—review & editing.
